# Genomic signatures of population isolation in an endangered European rodent

**DOI:** 10.1186/s12864-026-12950-1

**Published:** 2026-05-18

**Authors:** Paige A. Byerly, Alina von Thaden, Gregor Rolshausen, Berardino Cocchiararo, Stefanie Erhardt, Joanna Fietz, Alain C. Frantz, Eva Marie Kramer, Sarah P. Stubbe, Lorenzo Vinciguerra, Sven Winter, Johannes Lang, Holger Meinig, Sven Büchner, Carsten Nowak

**Affiliations:** 1 LOEWE Centre for Translational Biodiversity Genomics, Senckenberg Research Institute and Natural History Museum Frankfurt, Frankfurt, 60325 Germany; 2 Conservation Genetics Group, Senckenberg Research Institute and Natural History Museum Frankfurt, Gelnhausen, 63571 Germany; 3 German Zoological Society (DZG e.V.), München, 80469 Germany; 4 Institute of Biology, University of Hohenheim, Stuttgart, 70599 Germany; 5 National Museum of Natural History, Luxembourg, L-2160 Luxembourg; 6 Clinic for Birds, Reptiles, Amphibians and Fish, Working Group for Wildlife Research, Justus-Liebig-University Giessen, Giessen, 35392 Germany; 7 Naturmuseum St.Gallen, St. Gallen, 9016 Switzerland; 8 Faculty of Science and Technology, University of the Faroe Islands, Tórshavn, 100 Faroe Islands; 9 Senckenberg Biodiversity and Climate Research Centre, Frankfurt am Main, 60325 Germany

**Keywords:** conservation genomics, garden dormouse, habitat fragmentation, rodent genomics

## Abstract

**Background:**

The garden dormouse (*Eliomys quercinus*) is one of the fastest-declining mammals in Europe, and action is needed to prevent further population losses. The primary causes of declines are not well-understood, as the species experiences variable conditions and threats across its range, but likely include habitat fragmentation and loss. Previous genetic studies have provided evidence of highly structured garden dormouse populations in Western Europe, despite this region having been defined as a single clade with mitochondrial DNA analysis. Within Western Europe, the magnitude of declines has been recognized to be greater on the eastern edge of the species’ range, which could explain differentiation within the clade as resulting from diversity loss and genetic drift for regions under greater risk of extirpation. Here, we focus on fine-scale genomic differentiation across the Western European clade to explore the consequences of genomic erosion on the eastern region and to help identify mechanisms driving genetic differentiation within this species.

**Results:**

We found genetic differentiation both between and within major geographic regions. Populations located in the eastern edge of the species’ range showed stronger signs of population isolation, including structure between spatially distant populations, lower genetic diversity, and greater rates of inbreeding. However, all populations exhibited signals of recent rapid population decline. Outlier analyses indicated that differentiation between regions was primarily due to genetic drift resulting from isolation-by-distance rather than adaptive differentiation. We also found genetic structuring between populations within the Rhine Valley, despite apparent lack of physical barriers preventing dispersal among groups within this region.

**Conclusions:**

Our findings indicate that population isolation following habitat loss and fragmentation has likely been a major contributor to garden dormouse declines. Dispersal among disparate garden dormouse sampling regions is restricted—even across local spatial scales—leading to loss of genetic diversity and potential erosion of evolutionary potential. With 21st century declines expected to continue across the species’ range, even relatively common and well-connected populations are likely to follow the trajectory of the eastern populations, with increasing loss of diversity as populations contract and become more isolated.

**Supplementary Information:**

The online version contains supplementary material available at 10.1186/s12864-026-12950-1.

## Background

Small mammals (e.g., Rodentia, Eulipotyphla) are foundational to terrestrial food webs, and their conservation is crucial for maintaining ecosystem functioning [1–3]. Understanding patterns of contemporary decline and extinction in these groups is therefore critical for ongoing biodiversity conservation efforts. Small mammals have been characterized as less vulnerable to extinction than larger mammals, in part because they tend to be less persecuted, have smaller ranges, and exhibit higher fecundity [4–7]. However, such broad inferences are biased by research effort, as small mammals are understudied in comparison to larger-bodied, more charismatic taxa [8–13], meaning that these characterizations are based on limited data. Resulting data deficiencies from such research biases have critical effects on conservation planning. For example, in Europe, intensive management, together with changing governmental regulations and social attitudes, has helped reverse the wide-scale contemporary declines of large-bodied taxa such as carnivores, ungulates, and waterbirds [14–16]. By contrast, small mammals continue to decline in the 21st century [12, 17, 18]. However, most information on known population declines of small mammals in Europe is derived from anecdotal evidence or snapshot surveys rather than from data generated by rigorous long-term monitoring programs [12, 18, 19], giving us an incomplete picture of the factors driving these declines. Globally, over 85% of mammal species are small-bodied (< 5.5 kg), and almost half of these species are at risk [13]. Improving our understanding of population dynamics of small mammal species in response to factors such as landscape changes and anthropogenic disturbance thus represents a critical step forward in biodiversity conservation.

The garden dormouse (*Eliomys quercinus*), a nocturnal, hibernating rodent species of conservation concern in Europe, was classified as “vulnerable” by the IUCN in 2024 due to extensive population contractions and local extirpations [20, 21]. It has been characterized as the most severely declining rodent species in Europe, and is known to have disappeared from over 50% of its original range since the 1970s [20, 21]. Although the species is primarily associated with woodlands [22], it exhibits high rates of ecological plasticity across its range, occupying diverse habitats such as urban gardens and vineyards [23]. Accordingly, no single driver has been established as the primary cause of the garden dormouse’s sharp decline, although habitat fragmentation and loss [23, 24, 25], prey reduction [24, 25], rodenticides [26], and predation [27] have all been cited as contributing factors. Increasingly, climate change has also been implicated, with rising winter temperatures hypothesized to disrupt hibernation patterns [24].

Previous mitochondrial DNA (mtDNA) analysis defined the garden dormouse’s core central European range (Germany, France, Austria, Switzerland, Belgium, and Luxembourg) as a single clade [28], hereafter referred to as the Western European clade. However, our prior genomic work uncovered significant population structuring within this clade, including differentiation of individuals between the eastern and western boundaries [29]. A similar pattern of separate eastern and western lineages has been observed in the hazel dormouse (*Muscardinus avellanarius*), with the Rhine River representing an apparent barrier to dispersal between the two lineages in Germany [30]. Contrary to this pattern, our prior genomic analysis of the garden dormouse did not show a strong effect of the Rhine itself, with samples on both the eastern and western banks of the Rhine and its tributaries clustering as a single population [29], ruling out the Rhine as a possible vicariance factor.

Habitat conditions are variable within the Western European clade [23, 26], and could explain the differentiation between the eastern and western regions within the clade. Garden dormice on the eastern edge of the clade, which forms the easternmost boundary of the species’ range, typically inhabit spruce (*Picea abies*) forests in low mountain ranges, and are considered as a primarily forest-dependent species [26]. By contrast, garden dormouse adjacent to and west of the Rhine River are associated with urban habitats and lowland agricultural areas such as vineyards [23]. Climatic and habitat heterogeneity relating to factors such as temperature, precipitation, and elevation can be important drivers of positive selection and local adaptation in rodents, even at relatively small spatial scales [31–33], and could explain these differences. However, there are also known demographic differences between populations in the Western European clade [23]. Garden dormouse populations on the eastern edge of their range are observed to be isolated in highly fragmented habitat patches, while westerly populations have a more uniform distribution across the landscape and, presumably, a higher likelihood of dispersal among spatially-separated clusters [23]. This is significant because the contraction of garden dormouse populations across their entire European range appears to show an east-to-west pattern, with the species becoming increasingly patchily distributed with a reduced population density east of the Rhine River [21]. Given their mtDNA heterogeneity, differences in genetic variation between spatially isolated groups within the Western European garden dormouse clade could therefore be a modern phenomenon resulting from genetic drift [34–36] following habitat loss and fragmentation [37].

Conservation efforts for the garden dormouse are severely impeded by lack of data [19, 26]. Here, we expand on our prior genomic work [29] to investigate primary drivers of fine-scale genomic differentiation within the Western European garden dormouse clade. We theorized that any adaptive differentiation between sampling regions would be detectable through association of outlier loci with climactic variables. By contrast, if the primary causes of differentiation are related to genetic drift following declines and contractions, then we expected to see greater signs of isolation and genetic erosion in the eastern distribution of the species, where the garden dormouse has experienced more habitat loss and fragmentation than in the Rhine Valley. While garden dormice in the west of their range were established as a single group in our prior work [29], we additionally conducted an exploration of finer-scale population structuring within the western Rhine Valley region with the goal of helping identify the primary mechanisms driving differentiation within this species.

## Methods

### Sample collection

**Fig. 1:**
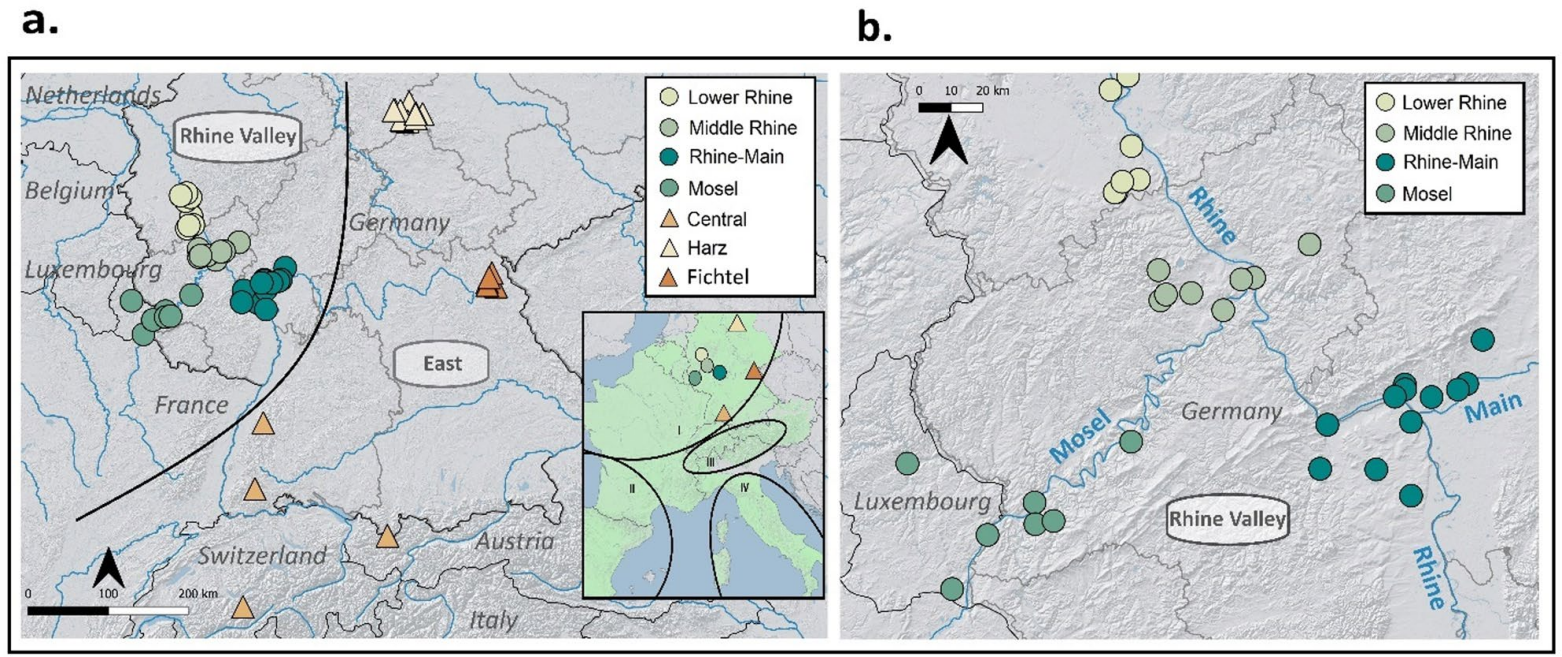
Distribution of samples within the Western European clade of the garden dormouse, with a priori assigned populations defined by colors and assigned region by shape (Rhine Valley [Lower Rhine, *n* = 10; Middle Rhine, *n* = 9; Rhine-Main, *n* = 7; Mosel, *n* = 14] as circles and East [Central, *n* = 6 ; Harz, *n* = 12; Fichtel, *n* = 5] as triangles) for (**a**) all analyzed samples (*n* = 63) and (**b**) within the Rhine Valley only (*n* = 40). The inset in a. represents the European range of the species, with numerals I-IV representing designated phylogenetic clades within this range, and with colors and shapes representing the sampling regions of this study within the Western European clade I

Samples were collected between 2011 and 2020 in the frame of a German-wide initiative for garden dormouse conservation (www.gartenschlaefer.de*)*, largely based on the contributions of citizen scientists [26]. Collection focus was on sample breadth, with a primary goal of collecting samples from each known garden dormouse population within the Western European clade (Fig. 1; Table S1). Samples (*n* = 63) were taken from salvaged individuals found during monitoring activities, from deceased animals recovered by rescue centers, and from museum collections.

### Genotyping

Tissue samples for were extracted using the DNeasy Blood & Tissue Kit (Qiagen). Extracts were quantified with fluorometry (Qubit, Thermo Fisher Scientific) and spectrophotometry (NanoDrop, Thermo Fisher Scientific) and screened for DNA integrity using gel electrophoresis to identify samples of sufficient quality for sequencing. Restriction site-associated DNA sequencing (RADseq) libraries from selected samples were prepared and sequenced at LGC Genomics GmbH (www.biosearchtech.com*)* [29]. Full details on enzymatic digestion, library preparation, and sequencing can be found in [38], but, briefly, libraries were prepared by digesting genomics DNA with the restriction enzyme MslI and ligating to inline barcodes, PCR-amplified, size selected for fragments > 300 bp using a Blue Pippin, and sequenced on a Illumina NextSeq 500/550. The raw sequencing data were trimmed using fastp v0.23.4 (Chen et al. 2018) with enabled base correction and low complexity filter to remove sequencing adaptors and poly(G) stretches at the end of reads. A 4 bp sliding window was employed to detect regions of poor quality (Phred score < 15). We removed reads if they fit into one of the following categories: read length below 36 bp, reads with > 40% low-quality bases, and reads with 5 or more undetermined bases (Ns). The trimmed reads were then mapped against haplotype 1 of the garden dormouse reference genome SAMN47866995 [29] with BWA-MEM v0.7.17-r1188 [39]. Mate coordinates in the resulting mapping files were filled in and the files were sorted and indexed using SAMtools v.1.18 [40]. Variant calling was performed with the ref_map.pl pipeline for reference-aligned reads in stacks v2.65 [41] with a minimum percentage of individuals per population of 80. Variant filtering was performed with VCFtools v0.1.16 [41] to remove indels, keep only biallelic SNPs with a depth per individual between 6 and 50, and remove the sex chromosomes. Samples were furthered filtered based on analysis type (Table S2).

Samples were a priori assigned to population by sampling location (Fig. 1a; Table S1). Given that we were interested in fine-scale population structure within the Rhine Valley region, we further assigned samples along the Rhine and its tributaries based on geographic sample clustering (Fig. 1a and b; Table S1), resulting in final assigned regional groupings of “East” (Harz Mountain region [hereafter, Harz], Fichtel Mountain region [hereafter, Fichtel], Central) and “Rhine Valley” (Lower Rhine, Middle Rhine, Rhine-Main, and Mosel). First- and second-order related individuals within each habitat group were then removed based on a priori population assignment and a relatedness threshold of > 0.10 using the King-robust method [42] as described in [29].

### Population structure

For all population structure analyses, we filtered our SNP set by removing samples with > 40% missing data and sites with > 25% missing data, requiring a minimum minor allele frequency (MAF) of 0.05, and selecting one random SNP selected per stack to minimize physical linkage disequilibrium (LD), which can bias genetic differentiation inferences (hereafter, the “LD-filtered” SNP set). We first visualized clustering within our data with principal components analysis (PCA) in the R package *adegenet* [42]. We ran PCA for both all samples combined and for the East and Rhine Valley sampling regions split into separate groups to visualize clustering within geographic regions. To check for the potential effects of non-random missing data on our results, we generated a subdataset with removal of individuals with > 25% missing data and sites with > 10% missing data and compared a PCA from this restricted dataset to that from the full dataset.

We used STRUCTURE v2.3.4 [43] within the program *Structure_threader* [44] to investigate admixture among populations and estimate the optimal number of genetic clusters *K*. We ran STRUCTURE with the admixture model and no prior population information, with an optimal burn-in of 10^4^ steps and 10^4^ additional steps, and for values of *K* = 1–7 with 10 replicates per iteration of *K*. We used the R package pophelper [45] to align runs, plot output, and determine the most likely value of K via both ΔK [46] and mean probability (MeanLn*P(K)*). We explored fine-scale variation in the Rhine by running the Rhine samples apart from the other regions, in addition to running all samples in a global analysis. We further investigated genetic differentiation among populations by calculating pairwise *F*_*ST*_ [47] between sampling locations in Stacks.

To determine if genetic structure within our dataset could be driven by geographic distance between populations, we tested for isolation-by-distance (IBD) by constructing an identity-by-state (IBS) distance matrix (1 − IBS) in SNPRelate 1.14.0 [48] and using this matrix and geographic distance between sample sites to conduct a partial Mantel test with 10^4^ permutations in ADE4 1.7.11 [49]. We visualized results as a two-dimensional kernel density estimation using the MASS package in R [50].

We further investigated patterns of spatial genetic differentiation in our data by analyzing spatial autocorrelation among samples using Moran’s eigenvector mapping (MEM) analysis in the R package MEMGENE 1.0.1 [51]. As in other spatial genetic analyses, MEMGENE uses Euclidean distance between samples to find correlations between spatial and genetic distance; however, it enables detection and analysis of nodes within the data set, with which weak and cryptic spatial neighborhoods of genetic differentiation can be visualized. MEMGENE produces eigenvector variables representing axes of spatially structured variation, which can be mapped onto sample coordinates, allowing visualization of primary patterns of genetic distance among samples [51]. Moreover, the spatial eigenvectors are regressed against genetic distances to evaluate the effect size (adjusted r²) of spatial variation on genetic structure [51]. We used MEMGENE to analyze spatial genetic patterns for both the entire sample set and for a subset of the Rhine Valley only, and plotted the first two variables for each spatial set.

### Demographic history

#### Effective population size

We estimated contemporary effective population size (N_e_) with the LD-filtered SNP set via currentNe v1.0 [53], which quantifies N_e_ using LD estimates and is robust to differences in sample size between populations. Given that the precision of N_e_ estimates derived from the LD methods is highly affected by missing data [54], we filtered SNPs prior to analysis by generating separate VCF files for each sampling region and then removing all missing data within each file. Even though this resulted in uneven numbers of SNPs between sampling regions, currentNe has been found to be robust to differences in SNP count if base count is above several thousand SNPs [55], and this discrepancy was therefore unlikely to strongly influence results. We analyzed N_e_ for each sampling site individually due to evidence for population structuring within our greater sampling regions, which can lead to biased N_e_ resulting from the Wahlund effect [56].

Recent (≤ 200 generations) trajectories of N_e_ were inferred using GONE [56]. GONE assumes that populations are closed and panmictic, and output can be biased when these assumptions are violated [56]. Results can also be influenced by low migration rates between population segments [57]. When we ran Harz and Fichtel as a single population, results showed patterns consistent with bias due to structure between the sampling regions resulting from low migration rates [57], and we therefore ran Harz as a single, separate population representing the East, omitting the Fichtel and Central sampling regions due to low sample size. For the Rhine River valley, we ran all samples from the Rhine Valley region as a single population. We ran GONE for 20 runs per population with the following parameters: Haldane correction for distance; 2000 generations with 400 bins (although only the first 200 generations are reported); MAF = 0.0; 24 chromosomes included (sex chromosomes omitted); max value of hc = 0.05; 40 reps (recommended); and a recombination rate of 0.75 cM/Mb.

#### Genetic diversity

Because estimates of genetic diversity can be biased by missing data, we filtered our full SNP set by removing individuals with missing data > 30% and variant sites with missing data > 10%. We then calculated individual observed heterozygosity (H_obs_) and the inbreeding coefficient F_IS_ using the --het function in PLINK [52]. We used t-testing in base R to check for significant differences in genetic diversity metrics between the East and Rhine Valley. In addition to these metrics of genetic diversity, we also looked for signatures of demographic changes, such as bottlenecks and inbreeding, via runs of homozygosity (ROH). Because of the difficulties inherent with reduced representation sequencing in estimating ROH, we do not present these methods in the main text, but have included them in the supplemental (Supplemental Methods).

#### Adaptive differentiation

Because all methods of outlier analysis can be biased by non-random missing data, we filtered the full SNP set for all outlier analyses by removing 5 individuals with > 30% missing data, resulting in a final set of 58 individuals. We additionally filtered samples by MAF = 0.05 to minimize the outsized effects of rare alleles on our output. We then used two uncorrelated methods to search for potential outlier loci and investigate the potential for adaptive differentiation between the East and Rhine Valley geographic sampling regions.

First, we identified candidate outliers as SNPs for which allele frequencies showed excessive differentiation along *k* principal component axes, an approach that is relatively robust towards continuous population structure and/or admixed individuals [58] with the R package *pcadapt* v4.0.3. We determined the optimal number of *k* principal components for the analysis with a screeplot and visualized SNP loadings for each selected PC to make sure that the output was not violated by linkage disequilibrium or other confounding factors. *P*-values for each SNP were transformed into *q*-values using the R package *q-value*, and SNPs with a conservative threshold of q ≤ 0.05, corresponding to a false discovery rate of 5%, were retained. These SNPs are assumed to make a larger-than-average contribution to the recovered population structure, and might thus be associated with adaptive outlier loci [58].

Second, we used partial redundancy analysis (pRDA) to identify outliers as SNPs with allele frequencies that correlated to environmental variables. While outlier analyses can result in false positives due to the confounding effects of neutral population structure and geographic distance, pRDA reduces these effects by using constrained (environmental) ordination while also controlling for variation due to population structure and geographic distance [59]. pRDA is largely insensitive to both low overall sample size [59] and to differences in sample size between sampling regions [60], which were both factors in our dataset. It also enables partitioning of effects from environmental variables as separate from latitude and longitude, which would otherwise be correlated with many climatic variables. We extracted environmental variables from individual sampling coordinates using WorldClim 2.1 [61] at 30-second (~ 1km2) resolutions in QGIS (QGIS.org 2024). We retained the environmental variables bio1 (Annual Mean Temperature), bio3 (Isothermality), bio5 (Max Temperature of Warmest Month), bio6 (Min Temperature of Coldest Month), and bio12 (Annual Precipitation), along with elevation. All environmental variables represent the average values from the years 1970–2000 and were standardized by mean.

Because pRDA requires no missing data, we imputed all missing data (median missing data rate of 0.08) within our SNP dataset based on the median allele frequencies for each sampling region. Following the best practices described in [62], we next ran a model of all environmental variables using the ‘rda’ function in *vegan* 2.6-4 [63] in R and used the ordiR2step function to compare the full model to a null model in order to select the best variables to retain using 1000 permutations and a significance of *P* < 0.01. To mitigate the effects of multicollinearity in our dataset, we then ran a model with just the retained environmental variables and removed those variables with a pairwise variance inflation factor (VIF) > 6, allowing for some moderate correlation. To approximate the role of population structure in our dataset, we ran a PCA on the filtered SNP set with no additional MAF correction and retained the first 3 PCs as proxies of neutral genetic structure. We used latitude (lat) and longitude (lon) of each sample’s collection location as proxies of geographic population structure (IBD).

In addition to detecting outliers, variance partitioning with pRDA can help parse out the contributions of different variables to driving genetic differentiation between sampling regions. We generated a global RDA model with all retained variables (variance ~ PC1 + PC2 + PC3 + lat + lon + bio1 + elevation) to investigate the effects of neutral genetic structure (drift), geographic distance, and environmental factors on total genetic variation within our dataset (as adjusted *r*^2^). To assess the individual effects of these factors we then ran three separate models: an environmental model (ENV: bio1 + elevation | lon + lat + PC1 + PC2 + PC3), a neutral population structure model (STR: PC1 + PC2 + PC3 + | lon + lat + bio6 + elevation), and a geographic distance model (GEO: lon + lat | bio6 + elevation + PC1 + PC2+ PC3). We tested all four models (global, ENV, STR, GEO) for significance with ANOVA and visualized distribution of genetic differentiation along variable axes with biplots. We defined the proportional effect of each model on overall genetic variation by subtracting the proportion of variance explained by each model by total variance. Because we were interested in hypothesized population structure within the Rhine Valley region, we reran these models following the same approach using samples from the Rhine Valley only.

Finally, we looked for the presence of outliers in the ENV model only for both the full sample set and the Rhine Valley sample set using the custom R function “rdadapt” [64] with a threshold value of q ≤ 0.05. We compared SNP loci identified as outliers between both the *pcadapt* and pRDA methods and identified ‘true’ outliers as those SNPs shared between both methods and retained a 20 bp window on either side of the outlier SNPs. We then visualized combined outlier SNPs against the full annotated garden dormouse genome [29] using the Senckenberg Research Institute’s mirror (https://genome.senckenberg.de/*)* of the University of California Santa Cruz (UCSC) genome browser [65] to investigate the potential of outliers in functional gene regions within 1,000-bp windows.

## Results

### Genotyping

Following alignment to a high-quality garden dormouse reference genome, SNP calling, quality control, and filtering, we obtained a total of 1,768,164 SNPs with a mean per site depth of 13.79**×** and mean frequency of missingness of 0.12.

**Fig. 2:**
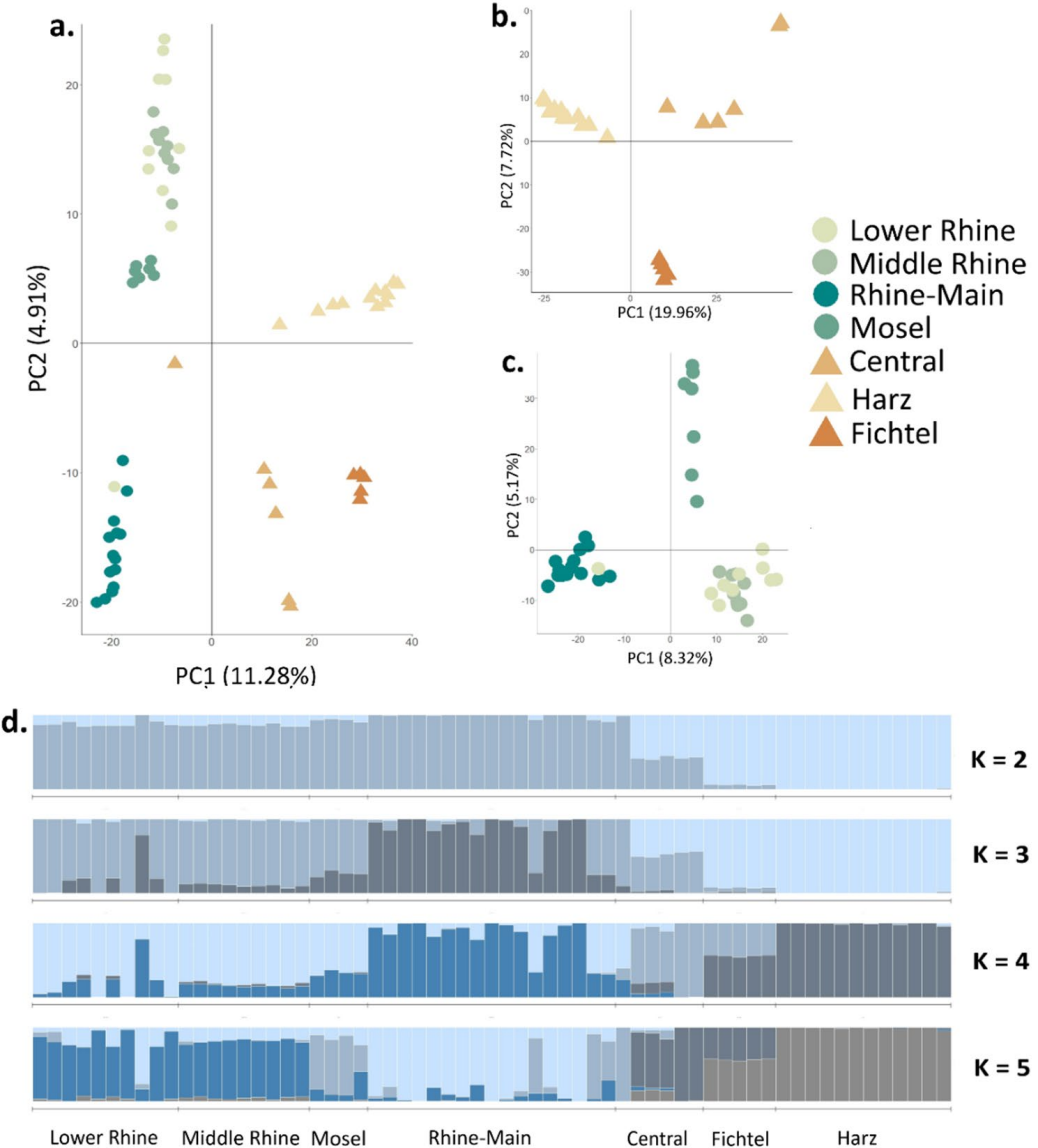
Genetic clustering inferred for the garden dormouse in Western Europe based on 59,974 SNP markers with: **a** principal component analysis (PCA) of samples from all analyzed populations (*n* = 63), with a priori assigned populations defined by colors and assigned region by shape (Rhine Valley as circles and East as triangles); **b** PCA within the East region only (*n* = 23); **c** PCA within the Rhine Valley region only (*n* = 40); **d** STRUCTURE for a priori clusters *K* ranging from 2 to 5 in, with x-axes representing geographic sampling location and y-axes the proportion of group membership

Population Structure Filtering for LD and MAF resulted in 59,974 SNPs for population structure analyses. Principal components analysis (PCA) showed a clear division between the East and Rhine Valley sampling regions on PC1, explaining 11.28% of the variation among samples, with samples further clustering by within-region groupings on PC2, explaining 4.91% of the variation (Fig. 2a). While most Rhine groupings overlapped, the Rhine-Main sampling region showed signs of differentiation on PC2. When analyzed separately by region, the East group showed strong differentiation between all three regions (Fig. 2b), while the Rhine split out into three clear clusters with the Lower and Middle Rhine groups grouping together (Fig. 2c).

One sample from the Lower Rhine clustered with the Rhine-Main group, but this was likely an artificially-translocated individual. Population clustering in the missing-restricted dataset matched that of the full dataset (Fig. S1), indicating that our clustering results likely reflected true differentiation within the samples, rather than biases resulting from missing data.

STRUCTURE results complemented PCA, with the likeliest number of clusters as *K* = 2 (Fig. S2). At *K* = 2, samples were strongly differentiated by the two main sampling regions west and east of the Rhine Valley (Fig. 2d). At values of *K* > 2, the Rhine-Main sampling region stood out as differentiated from the other Rhine Valley groups, and at *K* = 5 clusters were largely differentiated by region, with the East showing a cline structure likely driven by isolation-by-distance, but with the Rhine Valley separated into 3 distinct clusters (Fig. 2d). The fixation index *F*_ST_ was significant (a.k.a., confidence values did not cross 0) for all pairwise combinations between sampling regions (Table 1), and we found greater *F*_ST_ between pairwise combinations of the East and Rhine Valley regions.

**Table 1:** F_ST_ measure of genetic differentiation among pairwise groupings of garden dormouse from the Western European clade, with F_ST_ below the diagonal and 95% confidence intervals above. All values are significant (in that confidence intervals do not overlap 0)

	Central	Harz	Fichtel	Middle Rhine	Rhine-Main	Lower Rhine	Mosel
Central	—	0.23–0.24	0.09–0.10	0.13–0.14	0.12–0.13	0.12–0.12	0.16–0.17
Harz	0.23	—	0.16–0.17	0.21–0.22	0.24–0.25	0.20–0.21	0.25–0.26
Fichtel	0.17	0.17	—	0.18–0.18	0.19–0.20	0.17–0.17	0.21–0.22
Middle Rhine	0.16	0.21	0.21	—	0.07–0.10	0.02–0.03	0.10–0.10
Rhine-Main	0.15	0.24	0.22	0.07	—	0.06–0.06	0.09–0.09
Lower Rhine	0.15	0.21	0.20	0.03	0.06	—	0.08–0.09
Mosel	0.19	0.25	0.24	0.10	0.10	0.09	—

Mantel testing indicated significant isolation-by-distance (IBD; *r*^2^ = 0.81, *P* < 0.001). Visualization of the pairwise relationship between genetic and geographic distance showed that correlation densities corresponded to two distinct clusters within the East and Rhine Valley regions, with high IBD between regions (Fig. S3). In addition to IBD between regions, we found significant and similarly high IBD within both the East (*r*^2^ = 0.86, *P* < 0.001) and Rhine (*r*^2^ = 0.73, *P* < 0.001) regions, despite the smaller spatial scale for the Rhine Valley region.

**Fig. 3:**
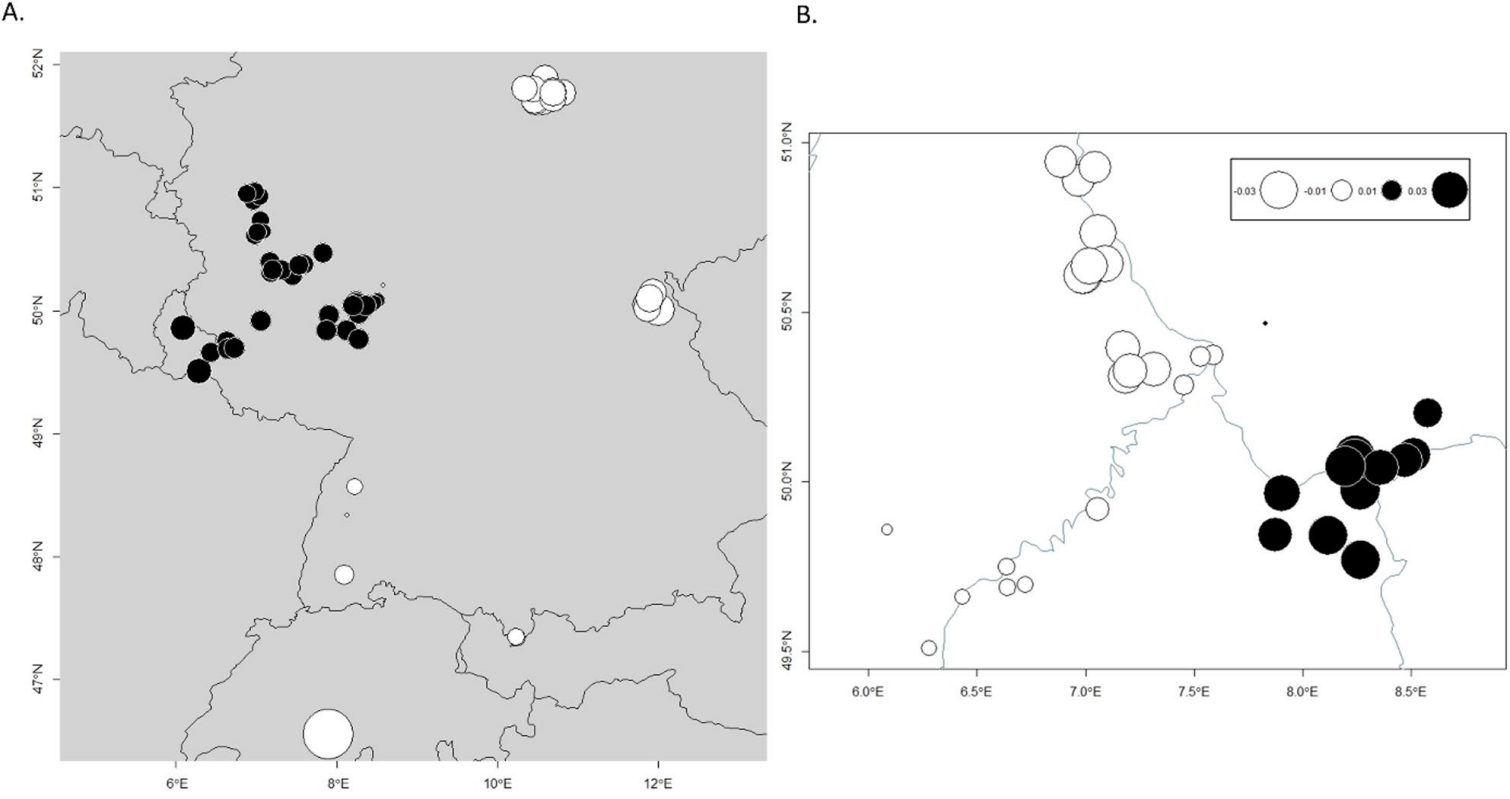
MEMGENE analysis of spatial relationships between (**a**) all samples and (**b**) the Rhine Valley only. Color and size represent individual MEM scores, with MEM scores representative of the proportion of genetic variance influenced by spatial distance. Similarity in size and color of circles indicate homogeneity of inferred genetic groups

The MEMGENE analysis of spatial patterns of genetic distance revealed patterns of variation at the scale of both the entire sampling region and the Rhine Valley region. For the full sample set, spatial patterns explained 12% of the genetic variation (adjusted *r*^2^ = 0.12), reflective of an overall pattern of spatial clustering with permeable barriers rather than pure IBD. Most variation was explained by the variable MEMGENE1 (48.8%) which was consistent with both PCA and STRUCTURE analysis in showing samples as delineated into East and Rhine Valley groupings, with the Central samples showing stronger relatedness to the Harz and Fichtel regions than to the Rhine Valley (Fig. 3A). MEMGENE2 explained 34.5% of the variation, and showed a similar pattern of east/west divide with the exception of a handful of outlier individuals within each sampling region (Fig. S4). For the Rhine Valley, less genetic variation was explained by spatial patterns (adjusted *r*^2^ = 0.06), indicating more permeability of barriers between spatial clusters, and more variation was represented by MEMGENE1 (48.4%) than by MEMGENE2 and 3 (26.6% and 22.9%, respectively). Visualization of MEMGENE1 showed (i) a dominant differentiation of the Rhine-Main from the other Rhine Valley clusters and (ii) a minor differentiation pattern between the western and the central/northern clusters—a pattern also picked up by PCA analysis (Figs. 2c and 3b).

Further inspection of MEMGENE2 suggests a certain permeability in north-south direction permitting a weaker scale of genetic pattern to develop between central and eastern clusters, rather than between western and eastern (or central) clusters (Fig. S5a, b).

### Demographic History

#### Genetic diversity

**Fig. 4:**
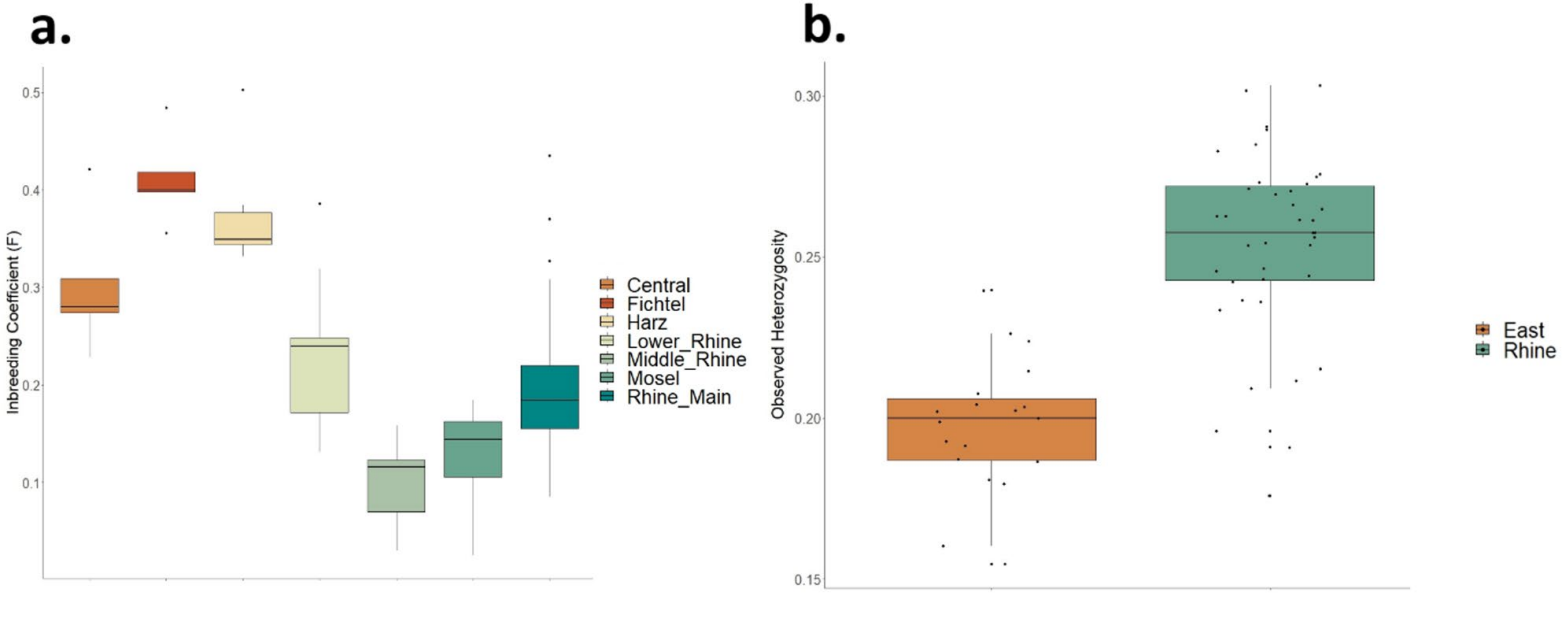
Differences in (**a**) the inbreeding coefficient FIS by sampling population and (**b**) observed heterozygosity for the Rhine Valley (Lower Rhine, Middle Rhine, Mosel, and Rhine-Main) and East (Harz, Fichtel, Central), based on 33,597 SNP markers

Inbreeding was greater for the East (FIS = 0.36, SD = 0.02) versus the Rhine (FIS = 0.18, SD = 0.02), with the difference between the two being significant (*P* < 0.001), and with the pattern observed across all analyzed populations (Fig. 4a). Similarly, we found significantly (*P* < 0.001) lower genetic diversity for the East (H_obs_ = 0.20, SD = 0.005) versus the Rhine (H_obs_ = 0.25, SD = 0.005; Fig. 4b). Estimates of inbreeding with ROH resulted in a comparable pattern (Supplemental Results). We found a greater mean proportion of genomic reads in ROH (F_ROH_; Fig. S6a) and ROH length (Fig. S6b) for the East as compared to the Rhine, indicating greater and more recent rates of inbreeding for the Eastern populations.

#### Effective population size

N_e_ varied greatly among sampling sites and showed no clear trend by sampling region (Table S3). The wide range of confidence intervals for all samples reflects a low degree of certainty in the results, which is likely related to the low sample size for all sites.

**Fig. 5:**
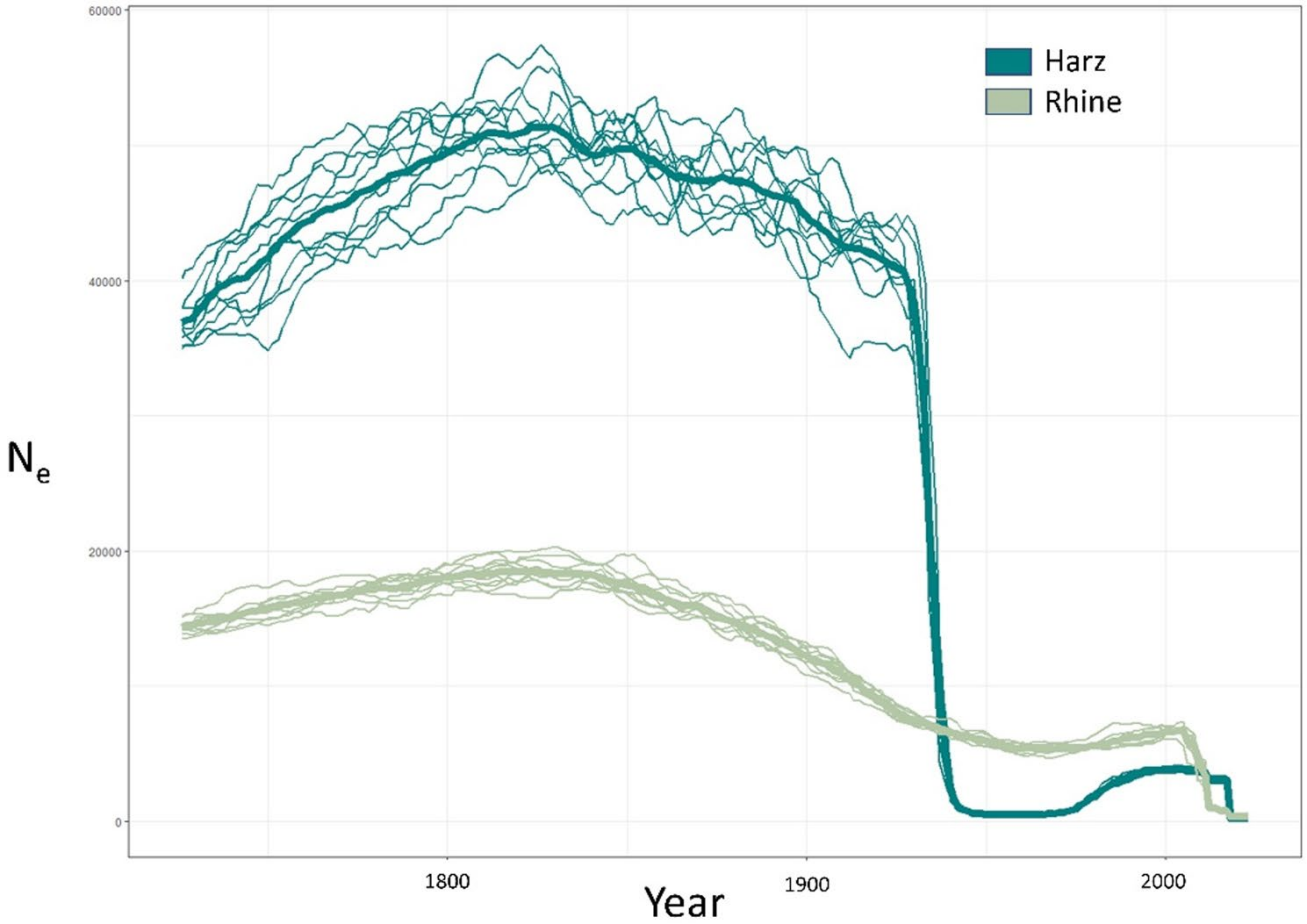
Inferred effective population size (N_e_) for the Rhine Valley (Lower Rhine, Middle Rhine, Mosel, and Rhine-Main) and Harz populations of the garden dormouse from approximately 1800 to the year of sampling. Year was estimated based on an assumed garden dormouse generation time of 1.5 years

For the Harz region, we observed an inferred N_e_ of approximately 40–60,000 until the early to mid-1900s, when the population exhibited a drastic and rapid decline (Fig. 5). By contrast, the Rhine region showed a lower but stable N_e_ with a peak of 20,000 in the early 19th century and with a more moderate decline through the 19th century (Fig. 5). Both regions exhibited a slight increase in N_e_ in the late 20th century followed by a rapid decline in the 21st century.

#### Adaptive differentiation

We retained three PCs for the *pcadapt* analysis (Fig. S7), from which 571 outlier loci were retained. For pRDA, variable selection identified bio1 (Annual Mean Temperature), bio5 (Max Temperature of Warmest Month), and elevation as variables to retain, all with *P* < 0.002. Of these, bio1 andbio5 had a VIF > 10, and we therefore retained only bio1 and elevation for the final environmental model.

**Fig. 6:**
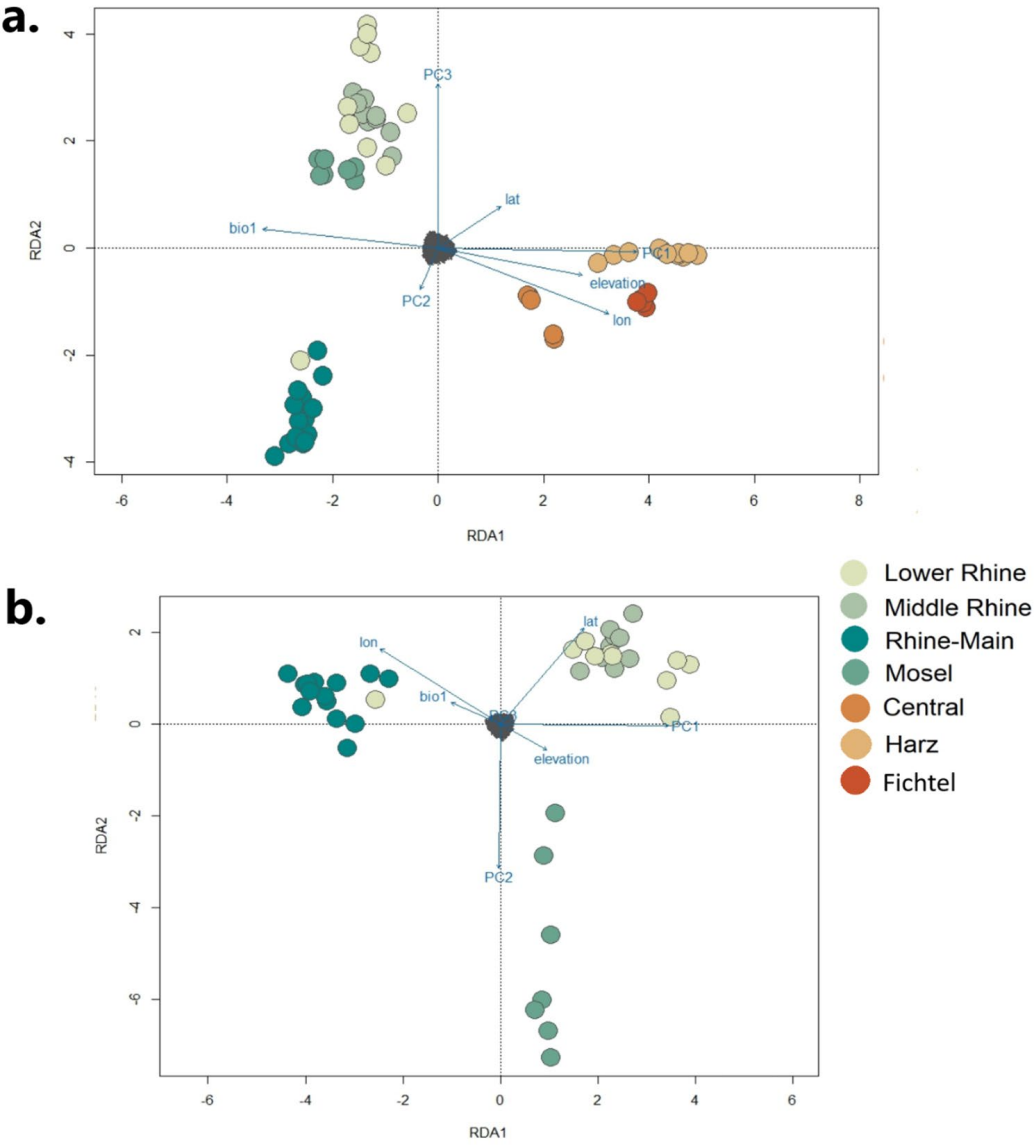
Plot of full RDA models with all retained variables representing geographic (“lat”: latitude, “lon”: longitude), neutral genetic (PCs 1,2, and 3), and environmental (“biol1”: average mean temperature, “elevation”: mean elevation at sampling location) factors explaining genetic distance between samples for (**a**) all samples and (**b**) the Rhine Valley only

The global model explained 26% of total variation in the full dataset. Samples were clustered by the East and Rhine Valley on RDA1 and were differentiated by the highly correlated variables of PC1, elevation, and longitude, all of which were negatively correlated to bio1 (Fig. 6a).

On RDA2, which was differentiated by PCs 2 and 3, the Rhine Valley samples were clustered by Rhine-Main and all other Rhine sampling regions (Fig. 6a). Model selection (Table 2) identified significant effects of neutral genetic structure and geographic distance on total genetic variation (8% and 5%, respectively), but not of environmental factors (3%; Fig. S8a-c). For the Rhine Valley region, samples were largely differentiated by sampling region, but with the Lower and Middle Rhine samples clustering together (Fig. 6b). Model results were similar to the full set (Table 2), with the global model explaining 26% of the variation in the SNP set and with variation significantly affected by neutral genetic structure and geographic distance (10% and 5%, respectively), but not environmental factors (2%; Fig. S9a-c). We detected 27 outlier loci differentiated by the ENV model, with outlier SNPs split relatively evenly between elevation and bio1 (Fig. S10). No outlier loci were detected for the STR model and GEO model or for any models in the Rhine Valley set. One locus (HAP1_SUPER_12:39560422) was shared between the pRDA and *pcaadapt* datasets, but was not located near any known functional sites on the annotated garden dormouse genome.

**Table 2:** RDA model results

Model	*r* ^2^	Df	*P*
Full sample set			
global (environment + structure + geography)	0.26	7	< 0.01
environment (average annual temperature + elevation)	0.03	2	0.41
structure (PCs 1–3)	0.01	3	< 0.01
geography (lat, lon)	0.05	2	< 0.01
Rhine Valley sample set			
global (environment + structure + geography)	0.26	7	< 0.01
environment (average annual temperature + elevation)	0.02	2	0.43
structure (PCs 1–3)	0.11	3	< 0.01
geography (lat, lon)	0.05	2	< 0.03

## Discussion

Although body size has been found inversely correlated to extinction risk in mammals [4–7], broader analyses have shown that smaller-bodied species may be more vulnerable to environmental and demographic stochasticity and therefore more susceptible to rapid collapse, particularly when populations are small or declining [66]. Here, we examined fine-scale population variation among spatially distinct Western European regions of the declining and endangered garden dormouse via reduced-representation sequencing. While demographic changes have been recorded across the species’ range, rigorous evidence of population declines and its genetic consequences has been lacking. Our findings validate inferences of accelerating declines in the 21st century, with our models of demographic evidence over time for both the eastern and western populations showing steep and rapid population drops in the early 2000s. Genetic diversity was consistently low across all populations we investigated, but particularly so for the small, isolated eastern populations, which showed evidence for high rates of inbreeding and low effective population sizes. Genetic variation was partitioned mainly on a broad-scale east-west basis, with geographic distance driving genetic differentiation among regions, as would be expected for such spatially distant populations of a small animal with limited dispersal capabilities. However, we also found genetic differentiation on a smaller scale, with some parts of the Rhine Valley also differentiated by spatial genetic clustering despite an apparent lack of physical barriers between groups. Our analyses indicate that dispersal among disparate garden dormouse sampling regions is severely restricted, resulting in an erosion of genetic diversity that is likely contributing to the species’ rapid decline.

### Phylogeographic population structure and genetic variance

The delineation of the eastern and western garden dormouse lineages into a single clade with mitochondrial [28], but not nuclear, DNA is indicative of a relatively modern split between these regions, given the faster mutation rate and smaller effective population size of mtDNA. Past mitochondrial analyses have suggested that garden dormice in the Western European clade emerged from a common glacial refugium following the last glacial period [28]. Further reconstruction of the demographic history from a high-quality garden dormouse reference genome indicated that the clade experienced a severe genetic bottleneck prior to this modern expansion [29]. This inferred history suggests that genetic diversity was low in Western Europe even prior to human disturbance. Indeed, more recent mtDNA analysis has suggested that this trait of low genetic diversity may be an intrinsic quality of the garden dormouse across its range [67]. From this reduced reservoir of genetic diversity, it then appears that population divergence between regions accelerated in modern times due to the amplification of drift effects in small populations [68]. We found that population divergence was greatest between the East and Rhine Valley regions, as evidenced by the higher *F*_ST_ between these broad regions; however, *F*_ST_ was still significantly high even between adjacent sampling regions within the Rhine Valley. MEMGENE and population structure analyses provided further support of regional differentiation in the Rhine Valley, splitting out the Rhine-Main sampling region from other Rhine Valley populations. This suggests that the Taunus Mountain range may be acting as a barrier to dispersal between the Rhine-Main and other regions, although it is also possible that the narrow Central Rhine Valley may instead be acting as an impediment to movement across the Rhine region. Hence, garden dormouse distributed across the Rhine Valley are not panmictic, but instead exhibit local-scale genetic differentiation.

We found no evidence that divergence between sampling locations was significantly associated with habitat variables, setting aside local adaptation as a main driver. Instead, our results suggest that reduced gene flow resulting from habitat loss and fragmentation significantly advanced independent drift dynamics, leading to further divergence. This is supported by our RDA models, which showed that genetic drift, as represented by neutral population structure, had the greatest effect on population differentiation, followed by geographic distance. Highly isolated wildlife populations exhibit lower levels of genetic diversity and higher rates of inbreeding than more well-connected populations, which are more likely to share dispersers [35, 37]. Our sampling scheme covers the full contemporary distribution of the garden dormouse in Germany [26], and our results therefore highlight the ongoing effects of isolation on the eastern German populations. Although a gradient of IBD would be expected for disparate garden dormouse populations, the extirpation of intermediate “stepping stone” populations has effectively cut the eastern populations off from sharing dispersers with other regions, leading them to become fully isolated. By contrast, the Rhine Valley population is adjacent to the larger, contiguous garden dormouse populations of France, and likely benefits from gene flow from these regions, a pattern that has also been observed in the European wildcat (*Felis silvestris*) [69]. With ongoing declines across the species’ range, even relatively common and well-connected populations are likely to follow the trajectory of the eastern populations, with continued genetic erosion as populations decline, contract, and become more isolated.

### Inbreeding and genetic diversity

Inbreeding between closely-related individuals promotes the spread and fixation of deleterious alleles within small populations and can reduce individual fitness when inbred individuals are subject to inbreeding depression [70–72]. We found greater inbreeding rates and lower heterozygosity for the East sampling regions, specifically Harz and Fichtel, which is consistent with our prior findings of lower genetic diversity for these sampling regions [29]. Although population dynamics can have unpredictable effects on F_IS_, greater prevalence of inbreeding is consistently associated with low N_e_ and can also result from severe recent bottlenecks [71]. The higher inbreeding rates in eastern garden dormice populations may reflect both known population declines and loss of genetic rescue resulting from dispersers from other populations, and are consistent with our detection of a bottleneck in the mid-1900s in the Harz region. By contrast, the lower inbreeding in the Rhine Valley sampling region may reflect the larger population size and greater connectivity within this region, as well as the less severe decline in the 20th century as reconstructed by GONE. Isolated populations of edible dormouse (*Glis glis*) have similarly shown greater rates of inbreeding and lower genetic diversity than larger, better-connected populations [73, 74], further pointing to the negative effects of isolation on dormice populations.

### Habitat fragmentation

Habitat loss and fragmentation are the primary drivers of extinction for terrestrial wildlife [75], and demographic changes following habitat loss can be rapid in small wildlife populations [76, 77]. Small, fragmented populations not only exhibit genetic erosion, but are also less resilient to environmental stressors, which can further exacerbate declines [78]. Europe is the most urbanized continent in the world, and only 1% of its contemporary surface can be considered as unaltered wildlife habitat [79]. Landscape changes on the continent have had major impacts on its terrestrial biodiversity, with associated declines for its wild species. In 2015, the range of the garden dormouse was estimated to have contracted by 51% since the 1970s, and landscape-level changes across its range have been implicated as primary drivers of this decline [21]. Isolation from range contractions is likely exacerbated by barriers such as roads and inhospitable matrices, as dormouse movement is inhibited by roads and areas without vegetative cover [80]. In our study, clustered garden dormouse populations in the east of their range represent isolated remnant populations of a formerly larger and more connected metapopulation [26]. Although population contractions and declines in the eastern part of the species range were first recorded in the 20th century, differences in genetic diversity parameters between the two regions suggest that cryptic declines may have been ongoing for centuries. The loss of genetic diversity through genetic drift following processes such as population isolation is a critical focus of conservation genetics. However, both simulated and in situ investigations of diversity loss have shown that the process of drift lags behind actual population declines, taking decades and even centuries to manifest as changes in genetic diversity [81, 82].

Reconstructions of changes in N_e_ over time via GONE show a sudden, steep decline in the eastern Harz region beginning in the 1930s–40s. The magnitude of the decline represented by GONE is more likely to be an artifact of population structuring in the dataset, possibly reflecting an abrupt change in migration such as severing of connectivity with a source population at the time of the apparent steep drop in N_e_ [57]. However, the trajectory is consistent with the theorized major change in the population’s demographics in the early to mid-20th century. By contrast, the Rhine population showed a steady, slow decline through the 20th century more consistent with what we would expect from a closed, panmictic metapopulation dynamic [57], which is predicted lead to a more gradual erosion of diversity. Both the Rhine Valley and Harz regions showed evidence for steep declines in the early 2000s, which matches best known records showing intensification of garden dormouse population collapses during this period, and is consistent with contemporary findings of high rates of inbreeding in both regions. Altogether, our results highlight multiple periods of declines within the Western European garden dormouse clade within the 20th and 21st centuries.

Conservation intervention is needed to halt the garden dormouse’s decline and prevent further population extirpations. Translocation of individuals between breeding populations has been effective in mitigating deleterious effects of inbreeding in other isolated wildlife populations [83, 84], and could be an option for enhancing genetic diversity within isolated garden dormouse populations. Because there is no evidence of local adaptation, there is no expectation that translocating individuals would cause problems due to outbreeding. However, while loss of genetic diversity and increased inbreeding is likely to harm populations in the long-term, introduction of individuals from larger populations risks introducing deleterious alleles into the east, further reducing fitness [85, 86]. Additionally, it is unclear if enhancing genetic diversity within populations would help stall or remediate garden dormouse declines. Primary causes of the species’ rapid declines across its range are still not fully known, limiting the effectiveness of conservation actions. Without a clear understanding of the factors directly contributing to declines of the garden dormouse in central and eastern Europe, translocation of breeding individuals becomes tantamount to moving dispersers from a source to sink population, and is unlikely to lead to long-term population growth. Conservation and research efforts should focus on identifying primary causes of regional-specific declines and work to address these in order to halt declines, after which translocation may be a viable option for enhancing long-term persistence of isolated populations when gene flow between isolated groups is not possible. Additional work is needed to identify barriers to dispersal, particularly in the Rhine Valley region, and more comprehensive sampling is needed in this region to identify landscape features that might be associated with restriction of dispersing individuals.

## Supplementary Information

Supplementary Material 1.

Supplementary Material 2.

## Data Availability

The raw genomic data supporting the conclusions of this article are available in the NCBI BioProject database (https://www.ncbi.nlm.nih.gov/bioproject/) under accession number PRJNA1142780. The scripts can be downloaded from https://github.com/pabyerly/TBG_GardenDormouse.
