# Supplementary material for: Genomic signatures of population isolation in an endangered European rodent
Source: BMC Genomics. 2026 May 18;27:483. doi: 10.1186/s12864-026-12950-1 (PMC13185252; doi:10.1186/s12864-026-12950-1)
Supplement: Supplementary file 1 — Supplementary Material 1. [file 12864_2026_12950_MOESM1_ESM.docx]

Supplemental Information

**Genomic Signatures of Habitat Fragmentation and Population Isolation in an Endangered European Rodent**

Paige A. Byerly^1,2^, Alina von Thaden^1,2^, Gregor Rolshausen^1,2^, Berardino Cocchiararo^1,2^, Stefanie Erhardt^3^, Joanna Fietz^3^, Alain C. Frantz^4^, Eva Marie Kramer^5^, Sarah P. Stubbe^5^, Lorenzo Vinciguerra^6^, Sven Winter^7,8^, Johannes Lang^5^, Holger Meinig^5^, Sven Büchner^5^, Carsten Nowak^1,2^

^1^ LOEWE Centre for Translational Biodiversity Genomics, Senckenberg Research Institute and Natural History Museum Frankfurt, 60325 Frankfurt, Germany

^2^ Conservation Genetics Group, Senckenberg Research Institute and Natural History Museum Frankfurt, 63571 Gelnhausen, German

^3^ University of Hohenheim, Institute of Biology, 70599 Stuttgart, Germany

^4^ National Museum of Natural History, L-2160 Luxembourg, Luxembourg

^5^ Justus-Liebig-University Giessen, Clinic for Birds, Reptiles, Amphibians and Fish, Working Group for Wildlife Research, 35392 Giessen, Germany

^6^ Naturmuseum St.Gallen, 9016 St. Gallen, Switzerland

^7^ Faculty of Science and Technology, University of the Faroe Islands, 100 Tórshavn, The Faroe Islands

^8^ Senckenberg Biodiversity and Climate Research Centre, 60325 Frankfurt am Main, Germany

**Supplemental Methods**

*ROH*

We compared genetic diversity and looked for signatures of both past and recent demographic changes, such as bottlenecks and inbreeding, via runs of homozygosity (ROH). ROH, are stretches of identical-by-descent segments in the genome formed when closely-related individuals interbreed. ROH represent excess homogeneity in the genome, and both the quantity and length of segments are informative with regard to both the approximate timing and severity of inbreeding [[1, 2]](https://paperpile.com/c/9K4raJ/eSehG+pMuoJ). While short read sequences derived from reduced representation sequencing methods may not be fully representative of ROH abundance across the genome due to the reduced fraction of the genome being represented by sequencing, such data can still be used to investigate inbreeding if SNP density is sufficiently high [[3]](https://paperpile.com/c/9K4raJ/7p26h). ROH analyses can be influenced by MAF and LD pruning [[4]](https://paperpile.com/c/9K4raJ/jOBln), and we did not filter SNPs used for ROH analysis for these factors. However, as the number of ROH segments has been found to be correlated to per-individual missingness in RADSeq datasets [[5]](https://paperpile.com/c/9K4raJ/b8mfO), we further filtered SNPs by first removing all individuals with missing data > 10% and then removing all variant sites with missing data. This resulted in a final SNP set of 105,847 SNPs and 49 individuals, with a mean SNP density of 55.75 SNPs/Mb, well above the minimum recommended density for rule-based ROH approaches [[3]](https://paperpile.com/c/9K4raJ/7p26h).

We then explored ROH abundance and length in our data using a rule-based approach in PLINK. We applied the homozyg algorithm using the following settings, which we customized based on our SNP density: window density of 50 SNPs, 1 heterozygote allowed per window, 1 missing call allowed per window, and ≤ 500 kb allowed between adjacent SNPs. A region was called an ROH if it fulfilled these criteria and was also ≥ 1000 kb in length, contained ≥ 50 SNPs, and had a minimum density of 70 SNPs per kb.

Longer ROH are representative of recent inbreeding, whereas short ROH reflect past demographic events [[6, 7]](https://paperpile.com/c/9K4raJ/j6QL8+NDDgQ), and we therefore used total length of ROH (L_ROH_) in centimorgans (cM) to approximate demographic histories and compare between sites. To estimate time since coalescence (defined here as the time point at which the two identical stretches of DNA originated in the same common ancestor), we calculated coalescence as number of generations back to the common ancestor *g* = 100/(2 $\times$ L_ROH_) [[8]](https://paperpile.com/c/9K4raJ/TTkFC). We converted *g* to number of years assuming a generation time of 1.5 years and Mb to cM assuming a recombination rate of 0.75 cM/Mb based on the corrected mean recombination rate estimated for brown rats [[9]](https://paperpile.com/c/9K4raJ/o4H70). As we did not call ROHs < 1 Mb, this capped our maximum detectable coalescence time at 66.67 generations or 100 years. To compare absolute ROH between sites, we calculated F_ROH_, or the proportion of the realized genome in ROH segments, as F_ROH_  = L_ROH_ /2500 Mb (the approximate size of the garden dormouse’s genome).

**Supplemental Results**

*ROH*

PLINK recovered 21,284 ROH segments > 1 Mb across the 49 samples analyzed. We found a greater mean number of total ROH fragments and proportion of genomic reads in ROH (F_ROH_) for the eastern sites as compared to the Rhine sites (Fig. S6a). Despite the greater mean F_ROH_ for the east (0.47) versus Rhine (0.39), mean length of segments L_ROH_ did not differ greatly between the east (2.48 Mb) and Rhine (2.40 Mb) regions (Fig. S6b), the average for both of which equates to approximately 17.92 generations or 26.90 years since the last common ancestor. L_ROH_ varied within sampling regions; however, the majority of fragments in both regions were relatively short (< 5 Mb), indicative of older inbreeding events or background relatedness. As in other studies [[10]](https://paperpile.com/c/9K4raJ/kwrX3), we found a strong association between the number of ROH fragments and L_ROH_ (*r*^2^ = 0.81).

While both the east and Rhine regions displayed some variation in length of ROH segments between sampling regions, all showed a mean peak between 3 to 4.5 Mb, reflecting a time to last common ancestor bounded within the late 1990s to mid-2000s. This is when declines were believed to accelerate for the garden dormouse in the Western European clade [[11]](https://paperpile.com/c/9K4raJ/t5asX), and ROH estimates are consistent with our GONE results showing declines in both the Harz and Rhine Valley sampling regions beginning around 2005. As ROH are broken up by recombination rate, and recombination rate is not known for the garden dormouse, our calculations to last common ancestor represent a rough estimate; however, recombination rates have been found to be consistently low in rodents [[12]](https://paperpile.com/c/9K4raJ/o4H70+u3RtW), and the actual genome-wide recombination rate of the garden dormouse is unlikely to vary significantly from our applied recombination rate of 0.77 cM/Mb.

We found greater abundance of ROH for the eastern sampling regions, specifically Harz and Fichtel, which is consistent with our prior findings of lower genetic diversity for these sampling regions [[13]](https://paperpile.com/c/9K4raJ/3T5xm). Although population dynamics can have unpredictable effects on ROH, greater prevalence of ROH is consistently associated with low N_e_ and can also result from severe recent bottlenecks [[7, 14]](https://paperpile.com/c/9K4raJ/MPe98+NDDgQ). The higher abundance of ROH in the east may reflect both known population declines and also loss of admixture from migration from other populations, and is consistent with our detection of a bottleneck in the mid-1900s in the Harz region. By contrast, lower abundance of ROH in the Rhine Valley sampling region may reflect the larger population size and greater connectivity within this region, as well as the less severe decline in the 20th century reconstructed by GONE.

Because we used reduced-representation sequencing, results from our genetic diversity and inbreeding parameters are not directly comparable to studies which use whole-genome sequencing to quantify ROH. However, we would expect a downward, and not upward, bias in ROH abundance with our sequencing strategy [[3]](https://paperpile.com/c/9K4raJ/7p26h), and our results may therefore underrepresent the magnitude of ROH in our sampling regions. Despite this, the mean F_ROH_ recovered for all garden dormouse sampling regions in this study (0.35) is on the higher end of ROH estimates among mammals and extremely high for rodents, which had a mean frequency of 0.09 among 12 species at a whole genome level [[15]](https://paperpile.com/c/9K4raJ/XeNMD). Given that ROH have been found to be positively correlated to body size, with lower average rates of ROH in smaller mammals due to generally high population abundances and reproductive rates [[15]](https://paperpile.com/c/9K4raJ/XeNMD+bblas), our results represent a low amount of genetic diversity for the garden dormouse.

**Supplemental References**

[1. Curik I, Ferenčaković M, Sölkner J. Inbreeding and runs of homozygosity: A possible solution to an old problem. Livest Sci. 2014;166:26–34.](http://paperpile.com/b/9K4raJ/eSehG)

[2. Gibson J, Morton NE, Collins A. Extended tracts of homozygosity in outbred human populations. Hum Mol Genet. 2006;15:789–95.](http://paperpile.com/b/9K4raJ/pMuoJ)

[3. Lavanchy E, Goudet J. Effect of reduced genomic representation on using runs of homozygosity for inbreeding characterization. Mol Ecol Resour. 2023;23:787–802.](http://paperpile.com/b/9K4raJ/7p26h)

[4. Meyermans R, Gorssen W, Buys N, Janssens S. How to study runs of homozygosity using PLINK? A guide for analyzing medium density SNP data in livestock and pet species. BMC Genomics. 2020;21:94.](http://paperpile.com/b/9K4raJ/jOBln)

[5. Duntsch L, Whibley A, Brekke P, Ewen JG, Santure AW. Genomic data of different resolutions reveal consistent inbreeding estimates but contrasting homozygosity landscapes for the threatened Aotearoa New Zealand hihi. Mol Ecol. 2021; December 2020:1–15.](http://paperpile.com/b/9K4raJ/b8mfO)

[6. Stoffel MA, Johnston SE, Pilkington JG, Pemberton JM. Genetic architecture and lifetime dynamics of inbreeding depression in a wild mammal. Nat Commun. 2021;12:2972.](http://paperpile.com/b/9K4raJ/j6QL8)

[7. Martin CA, Sheppard EC, Illera JC, Suh A, Nadachowska-Brzyska K, Spurgin LG, et al. Runs of homozygosity reveal past bottlenecks and contemporary inbreeding across diverging populations of an island-colonizing bird. Mol Ecol. 2023;32:1972–89.](http://paperpile.com/b/9K4raJ/NDDgQ)

[8. Thompson EA. Identity by descent: variation in meiosis, across genomes, and in populations. Genetics. 2013;194:301–26.](http://paperpile.com/b/9K4raJ/TTkFC)

[9. Dumont BL, Payseur BA. Evolution of the genomic rate of recombination in mammals. Evolution. 2008;62:276–94.](http://paperpile.com/b/9K4raJ/o4H70)

[10. Moran PA, Bosse M, Mariën J, Halfwerk W. Genomic footprints of (pre) colonialism: Population declines in urban and forest túngara frogs coincident with historical human activity. Mol Ecol. 2024;33:e17258.](http://paperpile.com/b/9K4raJ/kwrX3)

11[. Bertolino S. Distribution and status of the declining garden dormouse *Eliomys quercinus*. Mammal Review. 2017;47:133–47.](http://paperpile.com/b/9K4raJ/t5asX)

[12. Dumont BL, Payseur BA. Evolution of the genomic rate of recombination in mammals. Evolution. 2008;62:276–94.](http://paperpile.com/b/9K4raJ/o4H70)

[13. Byerly P, von Thaden A, Leushkin E, Hilgers L, Liu S, Winter S, et al. Haplotype-resolved genome and population genomics of the threatened garden dormouse in Europe. Genome Res. 2024;34:2094–107.](http://paperpile.com/b/9K4raJ/3T5xm)

[14. Hewett AM, Stoffel MA, Peters L, Johnston SE, Pemberton JM. Selection, recombination and population history effects on runs of homozygosity (ROH) in wild red deer (Cervus elaphus). Heredity (Edinb). 2023;130:242–50.](http://paperpile.com/b/9K4raJ/MPe98)

[15. Brüniche-Olsen A, Kellner KF, Anderson CJ, DeWoody JA. Runs of homozygosity have utility in mammalian conservation and evolutionary studies. Conserv Genet. 2018;19:1295–307.](http://paperpile.com/b/9K4raJ/XeNMD)

**Supplemental Figures**

**Figure S1.** PCA of population structuring from 33,597 SNP loci based on a SNP set filtered for >10% missing data.

**
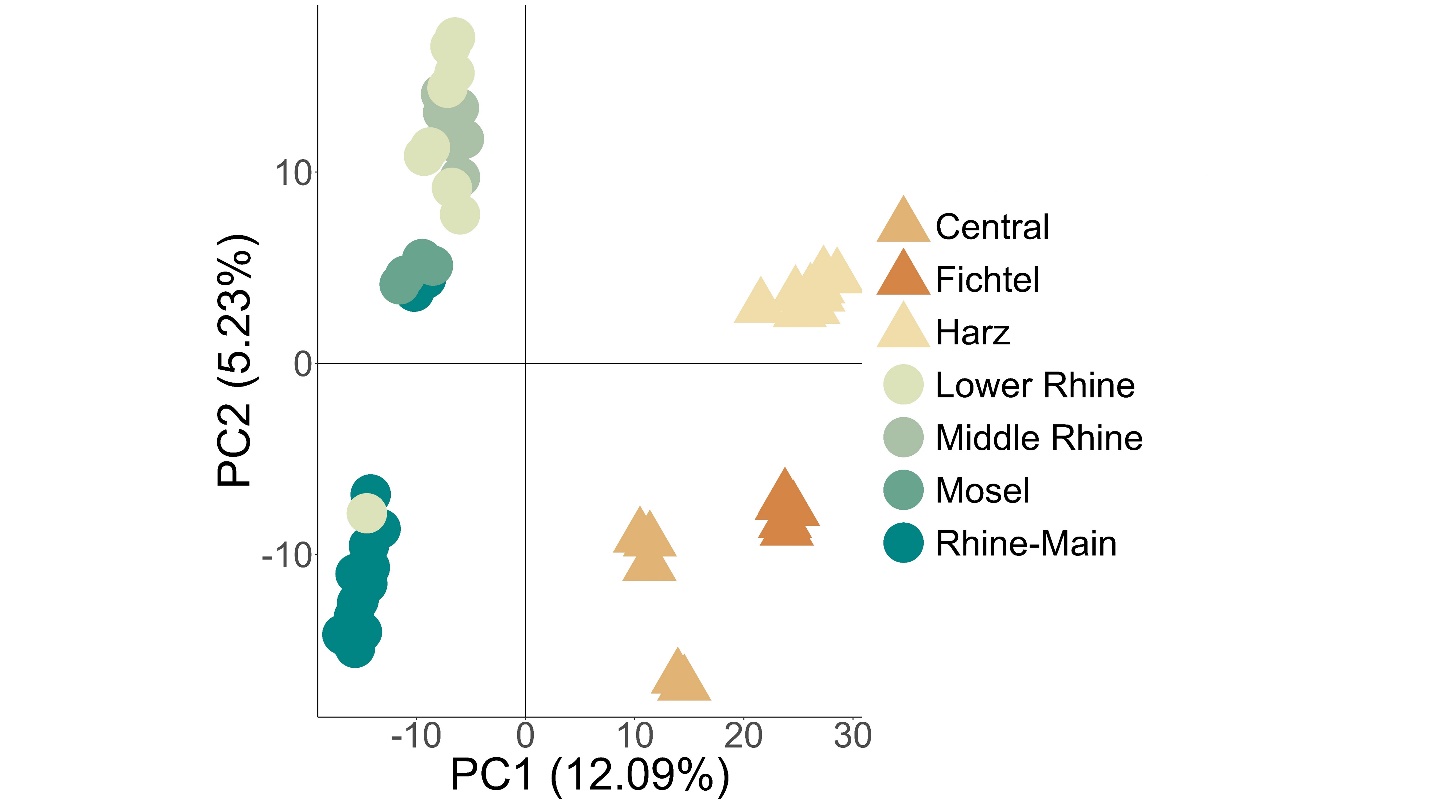
**

**Figure S2.** Structure output used for evaluating optimal likelihood values for *k* = 1–8 population clusters based on 59,974 SNP loci from n = 63 garden dormouse samples collected across Western Europe.


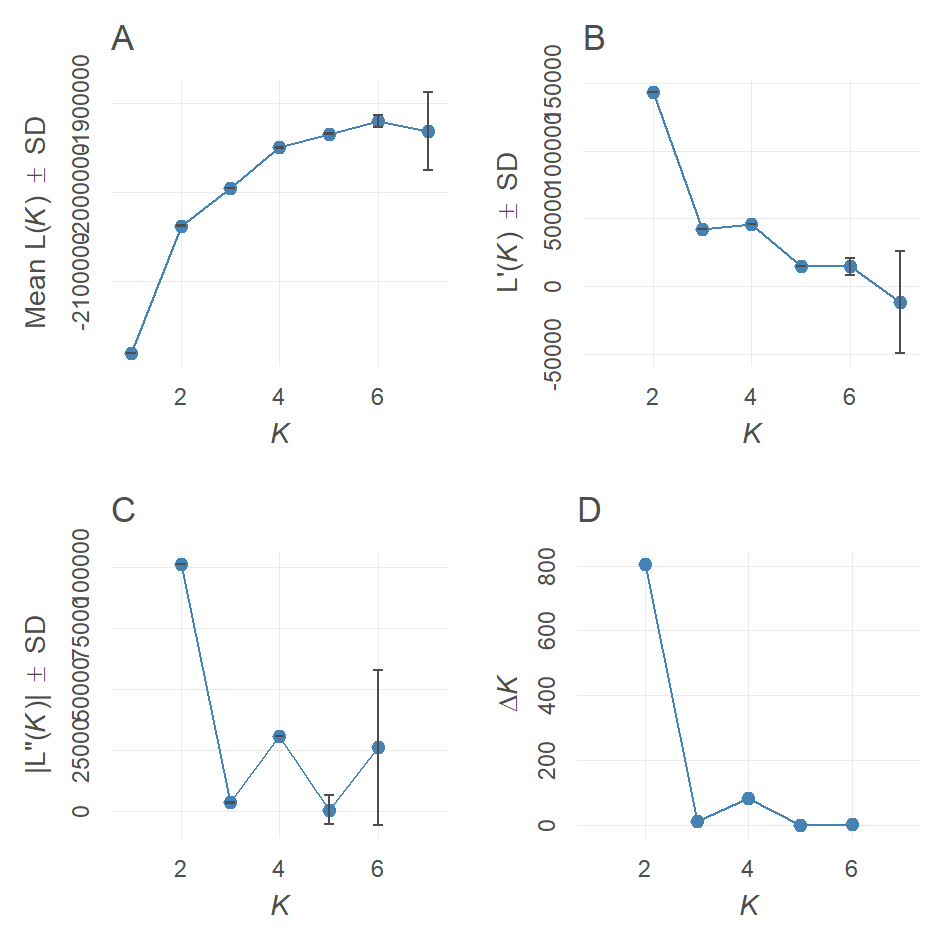


**Figure S3**. Kernel density plot testing for isolation-by-distance by evaluating correlation between geographic and genetic distance based on 59,974 SNP loci from n = 63 garden dormouse samples collected across Western Europe.


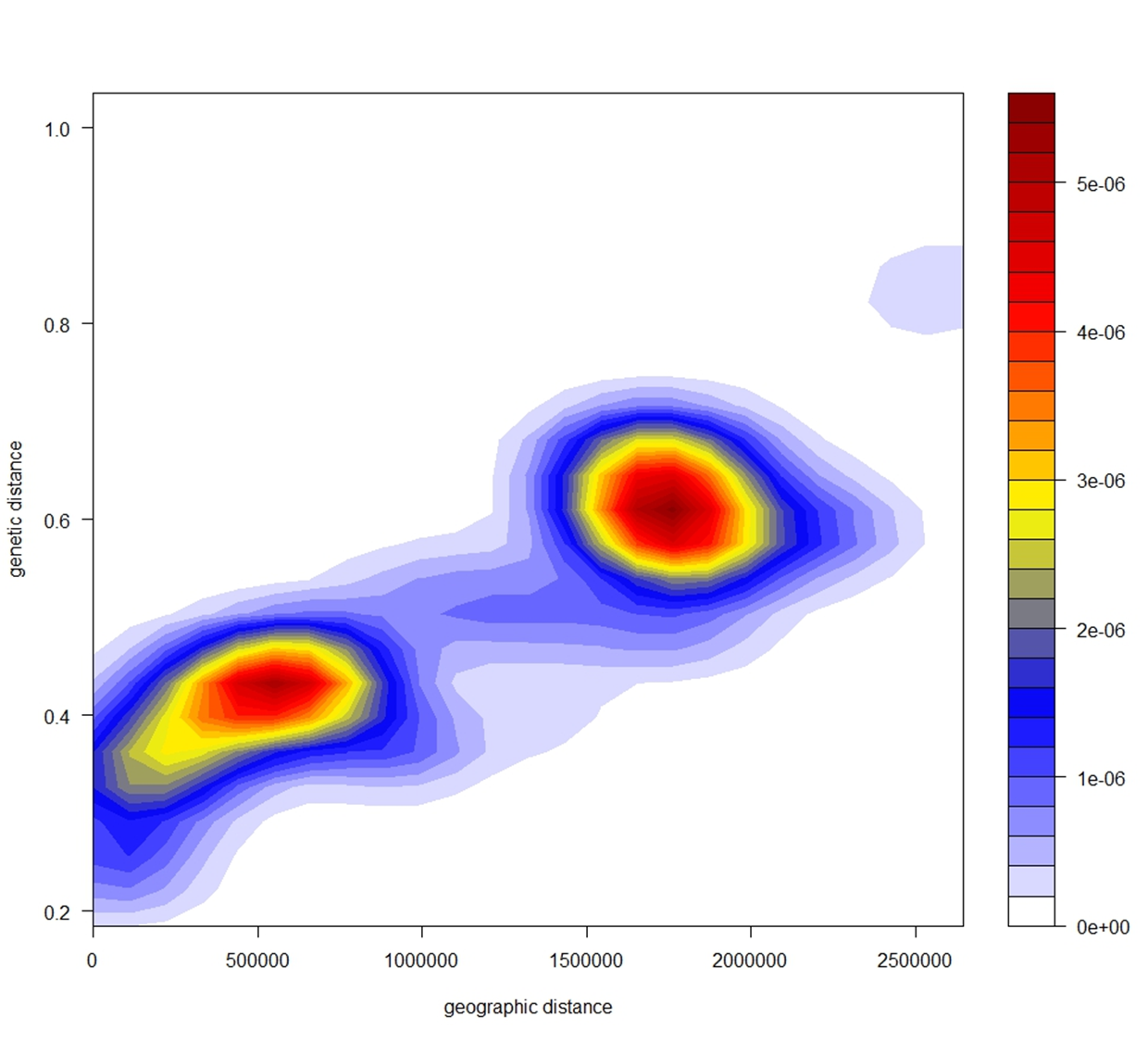


**Figure S4.** MEMGENE analysis of spatial relationships between a. All samples for the variable MEMGENE 2. Color and size represent individual MEM scores, with MEM scores representative of the proportion of genetic variance influenced by spatial distance. Similarity in size and color of circles indicate homogeneity of inferred genetic groups.

**
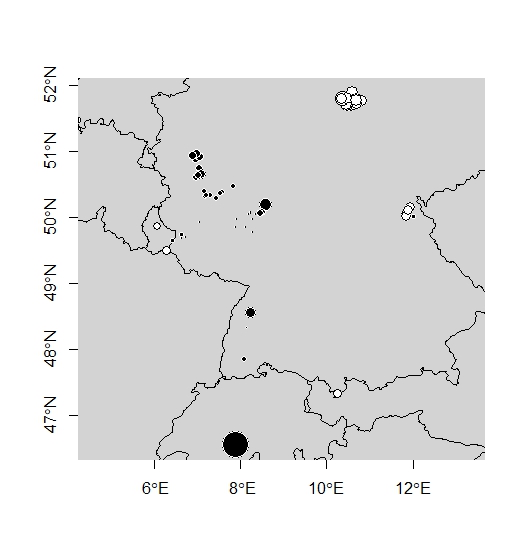
**

**Figure S5.** MEMGENE analysis of spatial relationships between a. the variable MEMGENE 2 and b. the variable MEMGENE 3 for the Rhine Valley only. Color and size represent individual MEM scores, with MEM scores representative of the proportion of genetic variance influenced by spatial distance. Similarity in size and color of circles indicate homogeneity of inferred genetic groups.


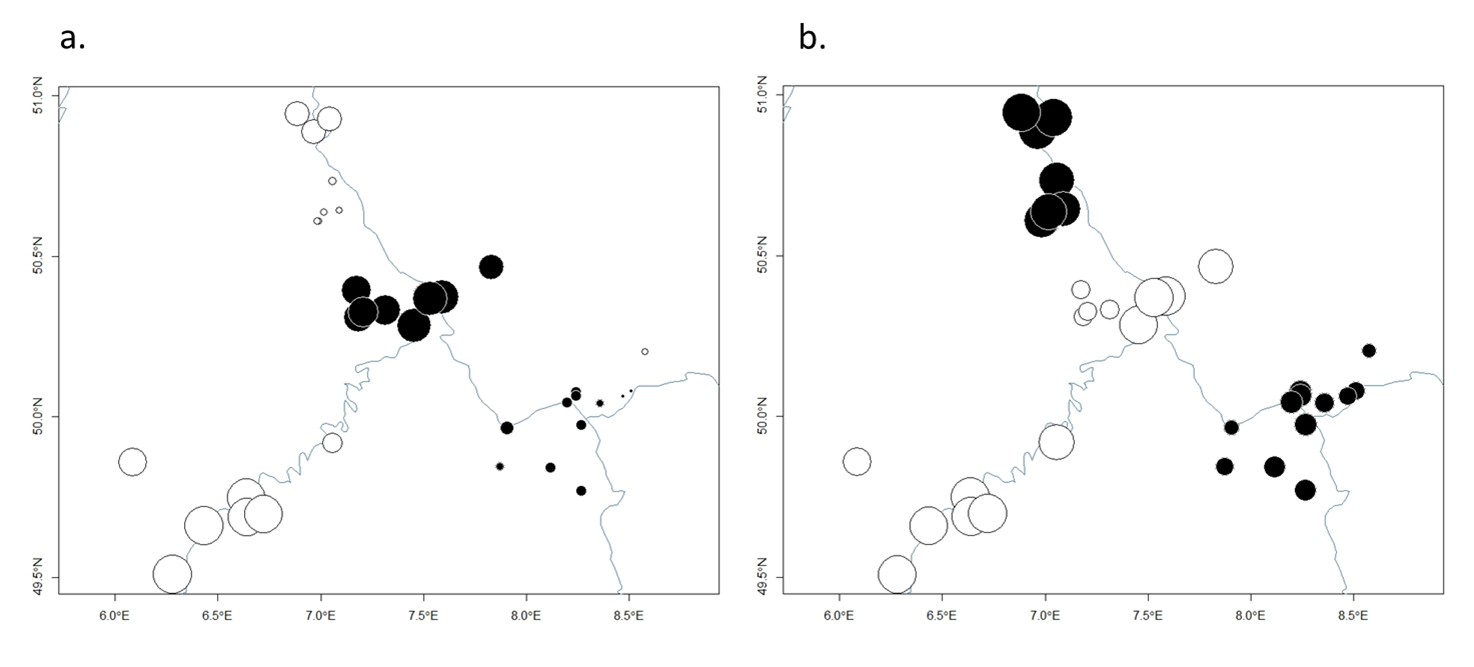


**Figure S6.** Graphic representation of runs of homozygosity (ROH) within the sampled garden dormouse a. proportion of the genomic reads in ROH: F_ROH_ segments (number of ROH) per sampling location b. ROH length by Kb (L_ROH_ [Kb]) by sampling

region.


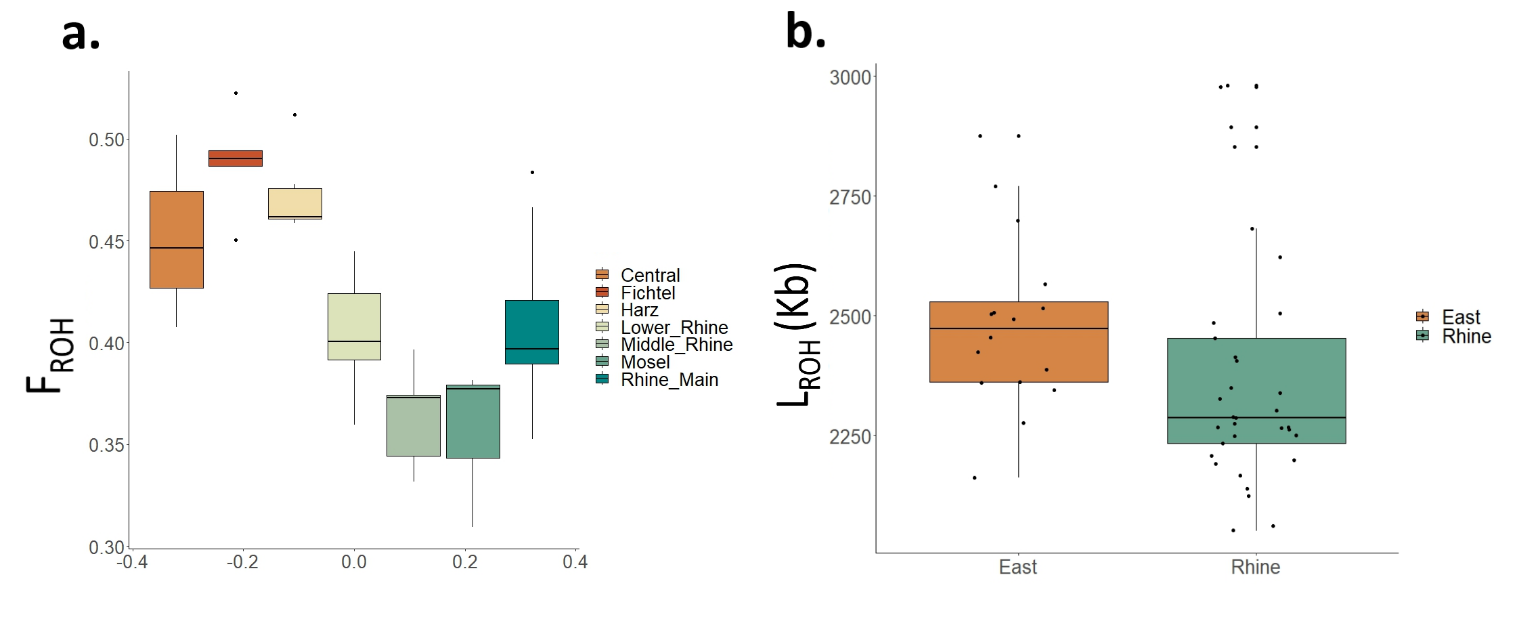


**Figure S7.** Scree plot to determine number of PCs to retain for outlier analysis with *pcadapt*.


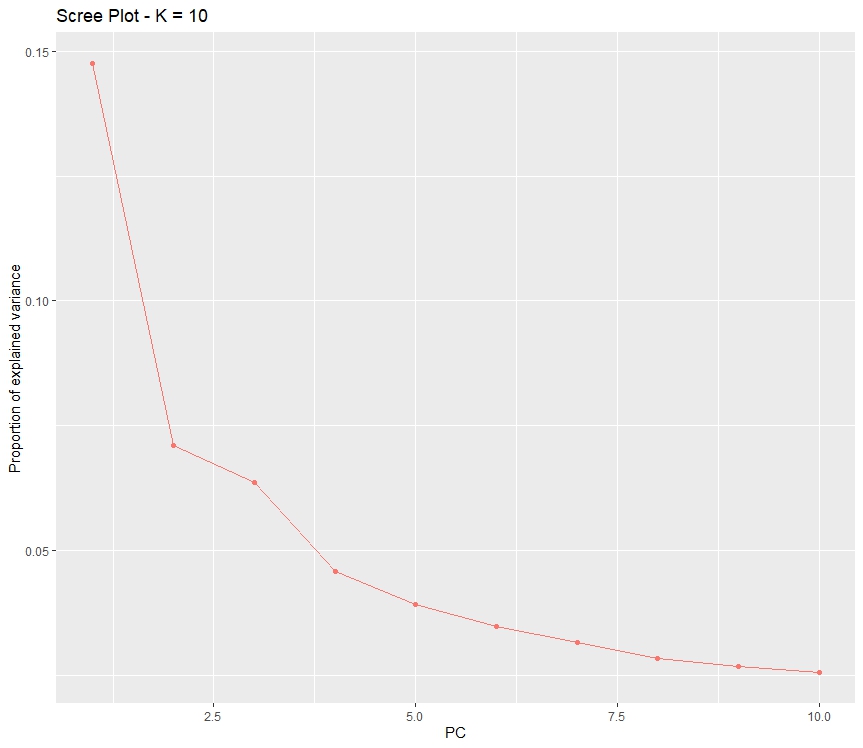


**Figure S8.** Plot of RDA models with all retained variables representing a. geographic, b. neutral genetic, and c. environmental factors explaining genetic distance between samples for all sampling regions.

**
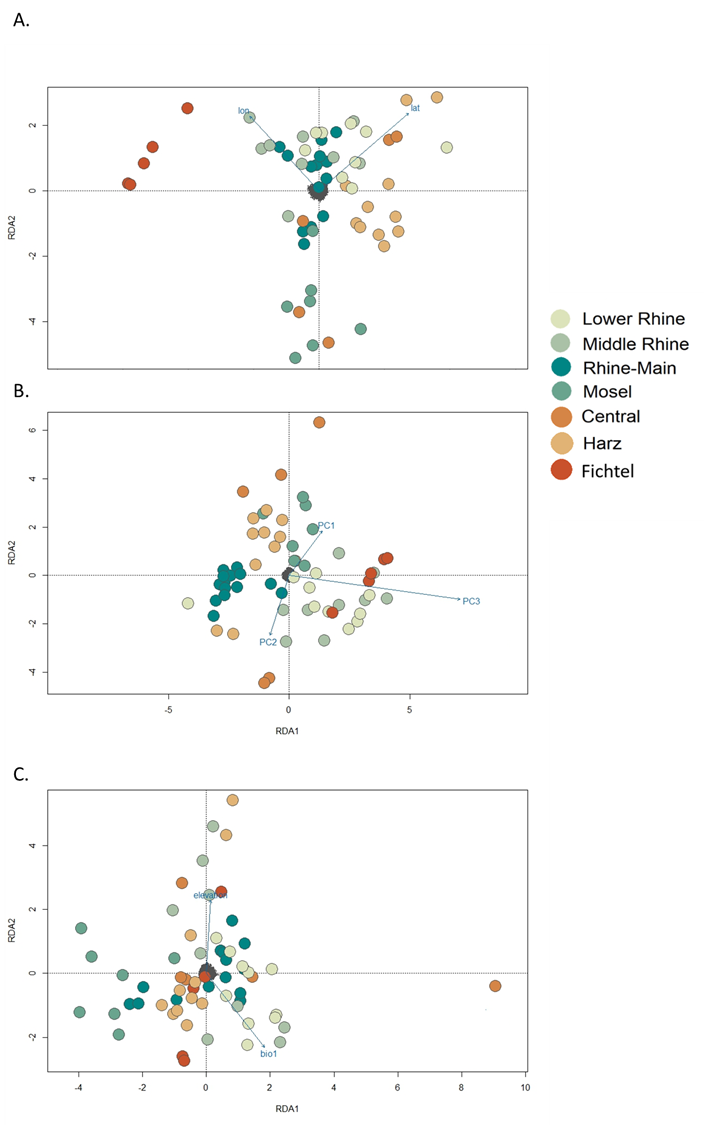
Figure Figure S9.** Plot of RDA models with all retained variables representing a. geographic, b. neutral genetic, and c. environmental factors explaining genetic distance between samples for the Rhine Valley only.

**
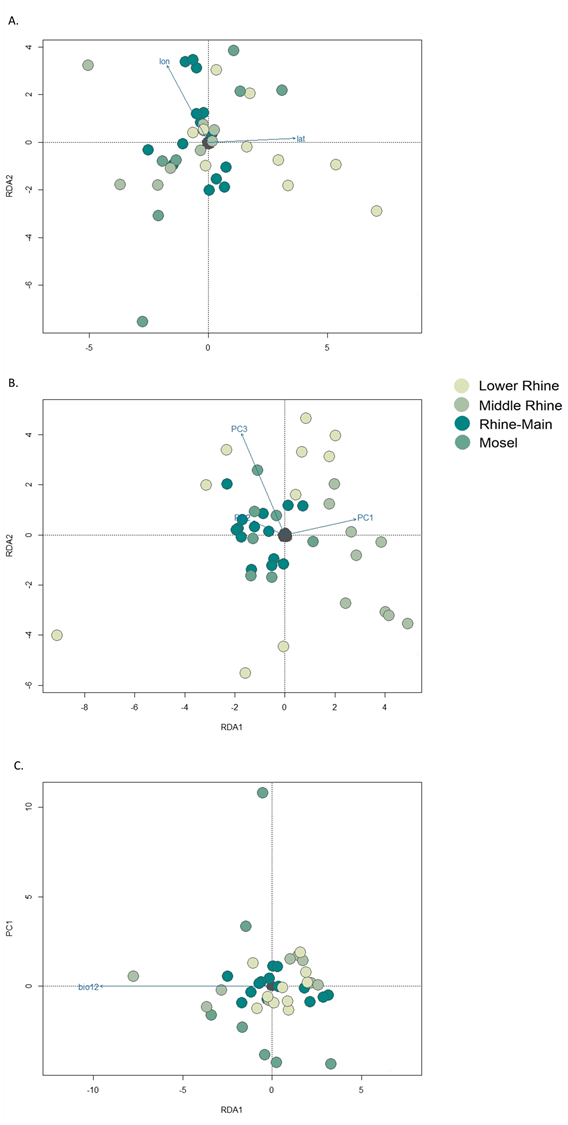
**

**Figure S10.** Placement of 27 outlier SNPs (in orange) derived from the environmental model for all sampling regions as differentiated by RDAs 1 and 2 within the RDA space.


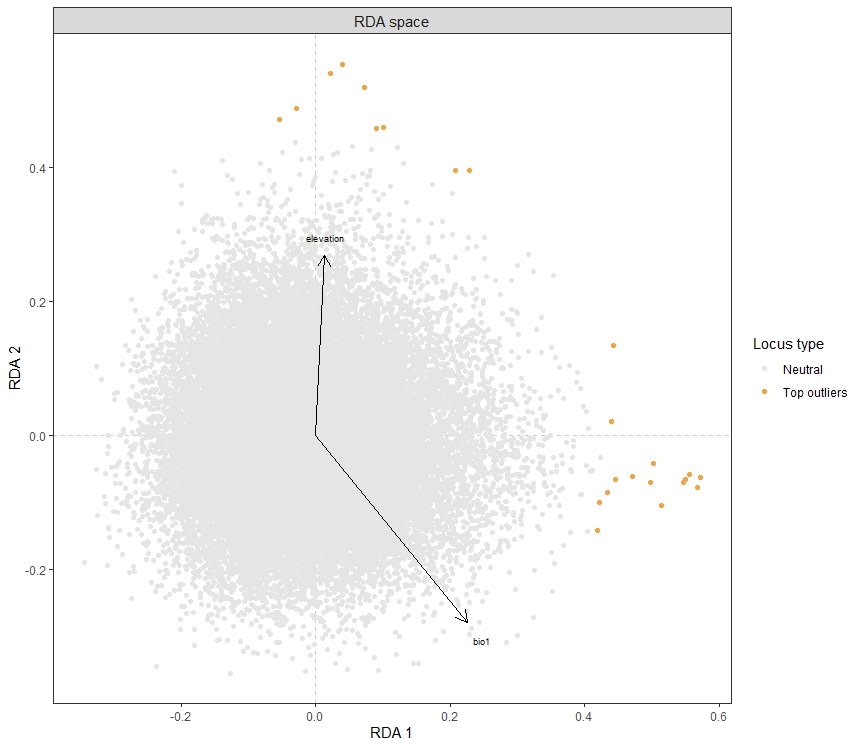


**Supplemental Tables**

**Supplemental Table S1.** Excel spreadsheet **“Supplementary_Table1”**

**Supplemental Table S2.** SNP sampling parameters and analyses type used for each SNP set.

| **Filtering Parameters** | **Analyses** |
| --- | --- |
| sites ≥ 25% missing, individuals ≥ 40% missing, MAF < 0.05, LD-filtering | PCA, STRUCTURE, IBD, MEMGENE |
| sites ≥ 10% missing, individuals ≥ 30% missing | Heterozygosity, Inbreeding coefficient |
| sites ≥ 100% missing, individuals ≥ 30% missing, MAF < 0.05, LD-filtering | Effective population size (N_e_), GONE |
| sites ≥ 25% missing, individuals ≥ 30% missing, MAF < 0.05 | outlier analysis, pRDA |

**Supplemental Table S3.** N_e_ estimates derived from currentNe (N_e_ estimation based on linkage disequilibrium between chromosomes) with number of individuals per population included in analysis (*n*), number of SNPs retained for each sampling region after filtering for missing data, and estimated N_e_

| **region** | ***n*** | **SNPs** | **Ne (90% confidence intervals)** |
| --- | --- | --- | --- |
| Lower Rhine | 10 | 25,566 | 35.96 (19.02, 67.99) |
| Middle Rhine | 9 | 26,530 | 58.28 (32.85, 229.39) |
| Mosel | 14 | 25.771 | 205.99 (81.05, 523.45) |
| Rhine-Main | 7 | 33,514 | 32.07 (14.22, 72.36) |
| Central | 6 | 14,102 | 4.18 (5.01, 6.52) |
| Northeast | 5 | 48,028 | 35.78 (11.62, 110.18) |
| Harz | 12 | 11,348 | 80.00 (36.34, 176.07) |
